# Impact of Complex Genetic and Drug–Drug Interactions on Tamoxifen Metabolism and Efficacy

**DOI:** 10.3390/jpm15110505

**Published:** 2025-10-23

**Authors:** Ibtissam Saad, Kaoutar Bentayebi, Soukaina Ettoury, Oumaima Zarrik, Ilhame Bourais, Saber Boutayeb, Caroline Samer, Youssef Daali, Rachid Eljaoudi, Sara Louati

**Affiliations:** 1 Research Laboratory in Drug Sciences, Mohammed VI Faculty of Pharmacy, Mohammed VI University of Sciences and Health (UM6SS), Casablanca 20100, Morocco; isaad@um6ss.ma (I.S.);; 2 Mohammed VI Center for Research and Innovation (CM6RI), Rabat 10112, Morocco; 3 Medical Biotechnology Laboratory, Medical and Pharmacy School, Mohammed V University, Rabat 10000, Moroccos.louati@um5r.ac.ma (S.L.); 4 Laboratory of Human Pathologies Biology, Department of Biology, Faculty of Sciences, Mohammed V University, Rabat 10000, Morocco; 5 Mohammed VI Faculty of Medicine, Mohammed VI University of Sciences and Health (UM6SS), Casablanca 20100, Morocco; 6 Faculty of Medicine, University of Geneva, 1205 Geneva, Switzerland; 7 Division of Clinical Pharmacology and Toxicology, Geneva University Hospitals, 1205 Geneva, Switzerland; 8 School of Pharmaceutical Sciences, Institute of Pharmaceutical Sciences of Western Switzerland, University of Geneva, 1205 Geneva, Switzerland

**Keywords:** pharmacogenetics, tamoxifen, breast cancer, tamoxifen metabolism, precision medicine

## Abstract

Tamoxifen remains the standard treatment for hormone-sensitive breast cancer. However, significant interindividual variability in treatment response is observed. This variability may be partially explained by differences in the biotransformation of tamoxifen, a prodrug, into its active metabolites. To address this, we conducted a comprehensive literature search across several databases to examine current evidence on single-gene and multi-gene variations throughout the metabolic and transport pathways of tamoxifen and their impact on pharmacokinetics and clinical efficacy. We also explore the influence of drug–drug–gene interactions and review clinical strategies currently employed to manage treatment variability. Overall, growing evidence highlights the influence of pharmacogenetic variability, particularly *CYP2D6* polymorphisms, on tamoxifen metabolism. Although its clinical use remains cautious and limited, a combined approach involving pharmacogenetic testing and therapeutic monitoring or phenotyping may help address treatment variability.

## 1. Introduction

Breast cancer remains the most prevalent cancer worldwide, with approximately 80% of cases classified as estrogen receptor-positive (ER+) [1]. Tamoxifen, a selective estrogen receptor modulator (SERM), is widely used as an endocrine therapy for hormone-sensitive breast cancer, significantly reducing the risks of recurrence, breast cancer-related mortality, and overall mortality [2,3]. Despite its proven efficacy, approximately 30% to 50% of patients experience relapse and disease progression, even among those with similar clinical profiles [4]. This observed variability in treatment response suggests that factors beyond standard clinical parameters may be involved. Notably, pharmacogenetic factors are increasingly recognized as contributors to treatment variability. These include drug–gene interactions (DGIs), which capture the influence of genetic polymorphisms on drug pharmacokinetics; drug–gene–gene interactions (DGGIs), which account for the combined effects of multiple polymorphisms in alternative pathways; and drug–drug–gene interactions (DDGIs), in which concomitant medications intersect with a patient’s genetic makeup [5].

DGIs occur when genetic polymorphisms influence the activity of drug-metabolizing enzymes (DMEs), leading to altered drug metabolism and potential variability in therapeutic response and the risk of adverse drug reactions (ADRs) [6,7]. In tamoxifen therapy, genetic variations in phase I enzymes impact the bioactivation of tamoxifen into its potent metabolites, such as endoxifen, while polymorphisms in phase II enzymes modulate metabolite clearance, and efflux and influx transporters regulate intracellular drug distribution. These genetic variations may contribute to differences in drug levels, potentially affecting treatment efficacy and safety.

Moreover, the complexity of these interactions is compounded by multifactorial influences, which may manifest as additive, synergistic, or opposing effects. Such variations encompass DGGIs, wherein mismatches between expected and actual metabolic capacities occur due to the genotype of alternative pathway enzymes. In the context of tamoxifen metabolism, the involvement of multiple pharmacogenes further accentuates the complexities surrounding treatment response.

Beyond genetic factors, tamoxifen metabolism can also be influenced by non-genetic elements. Drug–drug–gene interactions (DDGIs) arise when a patient’s genetic predispositions intersect with the use of certain concomitant medications. Even in the adjuvant setting, where tamoxifen is commonly prescribed, some co-medications may cause drug–drug interactions (DDIs). These interactions are generally well-recognized, routinely managed in clinical practice, and rarely result in serious consequences. Nevertheless, when combined with pharmacogenetic variability, even routine DDIs may have amplified effects, potentially altering tamoxifen metabolism and therapeutic outcomes. This is particularly relevant given that the vast majority of individuals harbor at least one clinically actionable genotype that could impact the treatment [8,9,10].

Advancements in pharmacogenomics have contributed to a better understanding of interindividual variability in drug response. However, despite growing interest and progress in the field, clinical findings remain heterogeneous, and the translation of pharmacogenomic insights into routine practice continues to face debate. This review examines the current body of evidence on DGIs, DGGIs, and DDGIs in the context of tamoxifen metabolism. Key enzymes and pathways are detailed, illustrating how pharmacogenetic variability and polypharmacy intersect to influence tamoxifen metabolism. Clinical challenges related to these interactions are considered, along with an overview of existing tamoxifen management strategies. The potential role of pharmacogenomic integration in clinical practice is also outlined, with a view toward supporting more individualized and effective therapeutic decisions.

## 2. Materials and Methods

A literature search was conducted using MEDLINE, Embase, and the Cochrane Library up to June 2025. Articles were included if they were original research studies or reviews reporting on genetic variability, comedication effects, or their impact on tamoxifen pharmacokinetics or clinical efficacy, and published in English. Additionally, references of included articles were checked to identify further relevant studies. Key information extracted included study design, patient population, genetic variants or metabolic pathways analyzed, comedication effects, pharmacokinetic data, and clinical outcomes. Findings were synthesized narratively.

## 3. Genetic Variation Impact on Tamoxifen Therapy

### 3.1. Tamoxifen Metabolism

**Figure 1:**
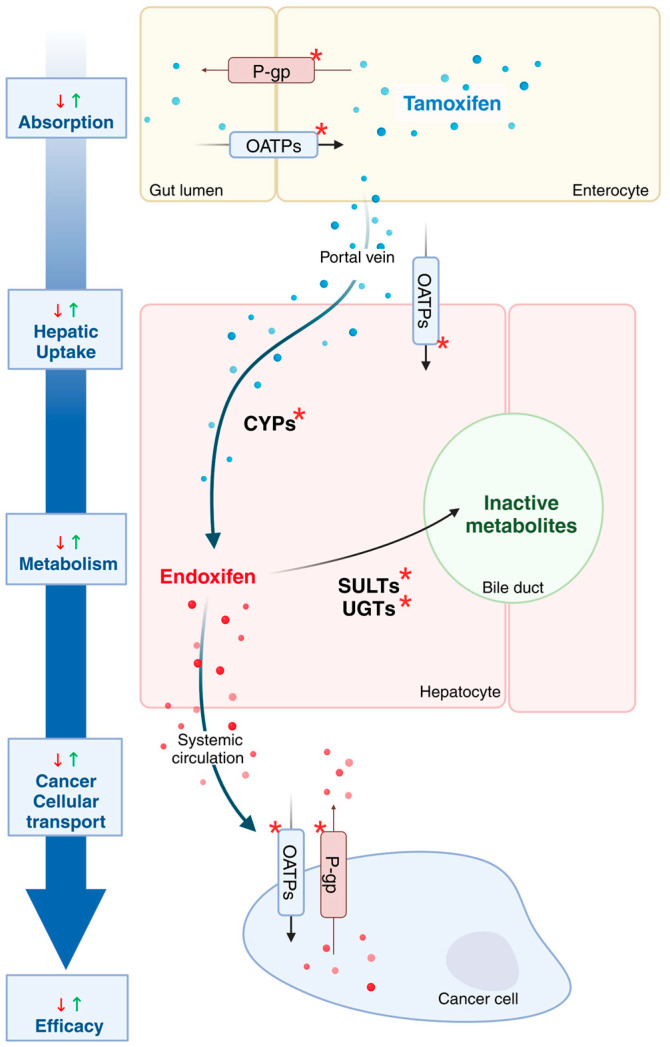
Pharmacokinetic pathway of tamoxifen and its clinical implications. After intestinal absorption mediated by transporters, tamoxifen undergoes hepatic metabolism through phase I and II drug-metabolizing enzymes, generating both active metabolites and inactive compounds destined for elimination in urine and bile. Active metabolites circulate systemically and reach their cellular targets, where they are imported by influx transporters and expelled by efflux transporters. Genetic polymorphisms and drug–drug interactions affecting drug-metabolizing enzymes or transporters can alter their function, disrupting the balance of activation, transport, and clearance, and thereby potentially modulating treatment efficacy. Sites marked with an asterisk (*) indicate points influenced by genetic polymorphisms or drug–drug interactions, which may change enzyme or transporter activity and affect tamoxifen efficacy or toxicity. The symbols ↓ and ↑ denote downregulation and upregulation, respectively. This pathway highlights potential key points that might underlie variability in treatment outcomes and, if proven clinically relevant, could guide future personalization of therapy. Created in BioRender (https://BioRender.com/p65t300, accessed on 28 February 2025).

Tamoxifen undergoes multiple metabolic and transport processes before exerting its therapeutic effect (Figure 1).

**Figure 2:**
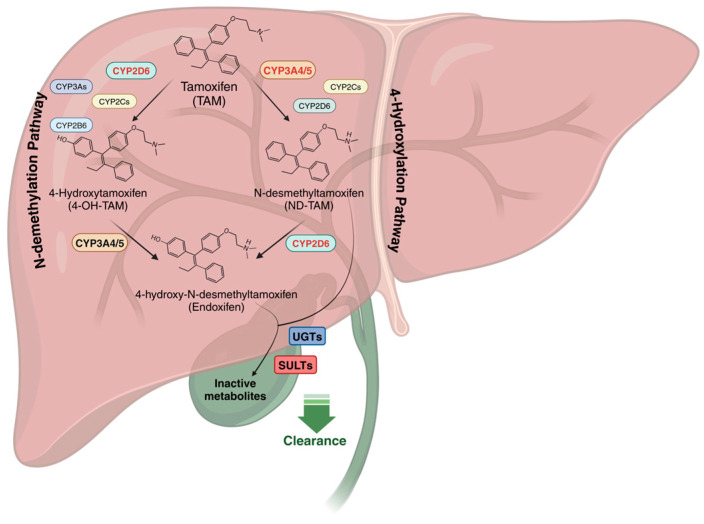
Hepatic metabolism and excretion of tamoxifen. Tamoxifen undergoes N-demethylation by CYP3A4/5 to form the major metabolite N-desmethyltamoxifen (NDM-TAM) and 4-hydroxylation by CYP2D6, CYP2C9, and CYP2C19 to produce the minor but highly active metabolite 4-hydroxytamoxifen (4-OH-TAM). Further metabolism of these intermediates, particularly by CYP2D6, generates endoxifen, the most potent metabolite. Phase II metabolism, mediated by UDP-glucuronosyltransferases (UGTs) and sulfotransferases (SULTs, especially SULT1A1), converts these metabolites into soluble conjugates excreted via bile and urine. Single or multiple genetic variations, together with drug interactions in one or more pathways, may influence metabolite levels and potentially affect therapeutic response. Created in BioRender (https://BioRender.com/p64b205, accessed on 28 February 2025).

As a prodrug, its efficacy depends largely on biotransformation by the cytochrome P450 (CYP) enzyme system, which generates active metabolites with up to 100-fold greater antiestrogenic potency than the parent drug (Figure 2) [11].

Tamoxifen metabolism predominantly proceeds through two key pathways, N-demethylation and 4-hydroxylation, both leading to the formation of the highly potent secondary metabolite, endoxifen. The N-demethylation pathway accounts for approximately 92% of tamoxifen biotransformation, primarily catalysed by CYP3A4/5. This results in the production of N-desmethyltamoxifen (DM-TAM), the most abundant metabolite, which is subsequently converted to endoxifen through CYP2D6. In parallel, a smaller fraction of tamoxifen undergoes 4-hydroxylation, mediated by various CYP enzymes, including CYP2D6, CYP2C9, CYP2C19, CYP2B6, and CYP3A4/5, generating 4-hydroxytamoxifen (4-OH-TAM) (refer to Figure 2). Although 4-OH-TAM is also a potent metabolite, its concentration is 10 times lower than that of endoxifen [12]. This metabolite is further demethylated by CYP3A4/5 to produce endoxifen, which is considered the primary active metabolite responsible for tamoxifen’s antiestrogenic effect [13].

Following this metabolic activation, tamoxifen and its active metabolites are converted into inactive, water-soluble forms through phase II metabolism. This process involves sulfate conjugation and glucuronidation, facilitated by phase II liver enzymes such as UDP-glucuronosyltransferases (UGTs) and sulfotransferases (SULT1A1/2). The transport of tamoxifen and its metabolites across various compartments is mediated by organic anion-transporting polypeptides (OATPs) for cellular uptake and expelled by P-glycoprotein (P-gp) transporters [14] (refer to Figure 1).

Although tamoxifen remains the gold-standard treatment for ER+ breast cancer, its metabolism and therapeutic response exhibits significant interindividual variability [15]. This variability raises concerns regarding the impact of polymorphisms in DMEs and transporters on treatment efficacy and clinical outcomes [16]. Genetic variations affecting the activity of these enzymes can lead to differences in the concentration of active metabolites, potentially affecting therapeutic success.

### 3.2. Drug-Phase I Metabolizing Enzyme Interactions

CYP2D6 is the primary enzyme responsible for metabolizing tamoxifen. Over 100 unique *CYP2D6* alleles have been identified, where 80% are associated with reduced or absent enzyme activity [17], according to the activity score system developed by Gaedigk [18]. Differences in CYP2D6 function can alter the metabolic conversion of tamoxifen. Patients with impaired CYP2D6 activity, such as poor metabolizers (PMs), tend to have lower endoxifen levels, potentially reducing the drug’s efficacy [7]. For instance, studies have demonstrated that women with the PM phenotype experience worse outcomes compared to those with normal metabolism across different settings, including premenopausal women of different ethnic backgrounds and postmenopausal women [19,20,21]; early-stage and advanced breast cancer [19,22]; metastatic disease [23,24]; and familial breast cancer [25]. In contrast, ultra-rapid metabolizers (UMs), who possess enhanced CYP2D6 activity, typically achieve higher endoxifen concentrations and may show a more favourable therapeutic response. For example, one study found that UM patients had a greater reduction in mammographic density compared to PMs, suggesting a more effective therapeutic outcome. However, UMs may experience stronger adverse effects due to higher levels of active metabolites, sometimes leading to treatment discontinuation [26]. On the other hand, PMs tend to experience fewer severe hot flashes [27], though they are more susceptible to other toxicities [28]. Additionally, some genetic variations, such as the reduced function CYP2D6*41 allele, have been linked to a higher incidence of certain side effects, like fatty liver, particularly among postmenopausal women on tamoxifen [29].

The relationship between *CYP2D6* genotype and tamoxifen treatment efficacy has been extensively studied. Endoxifen plasma concentrations are considered a potential predictor of therapeutic response [30]. Thresholds above 5.2 ng/mL (or 5.9 ng/mL in some studies) have been associated with a significantly reduced risk of relapse [31,32]. *CYP2D6* genotype can account for up to 54% of the observed variability in endoxifen levels [33], with even greater variability (135%) observed in premenopausal women compared to postmenopausal [34]. A recent genome-wide association study (GWAS) further reinforced the central role of *CYP2D6* by demonstrating a strong association between endoxifen concentrations and chromosome 22, where *CYP2D6* is located [35]. While *CYP2D6* genotype remains the most clinically relevant factor, variants in nearby genes that are in strong linkage disequilibrium with *CYP2D6* indicate that additional genetic elements within this chromosomal region may also contribute to interindividual variability in tamoxifen metabolism [35,36].

Despite the strong biological rationale, clinical evidence remains mixed. Some studies have reported clear associations between *CYP2D6* genotype and tamoxifen outcomes, while others found no significant impact [37,38,39,40,41,42,43,44]. These inconsistencies may stem from several factors, including small sample sizes, incomplete genotyping and heterogeneity in genotyping panels, use of somatic rather than germline DNA, co-administration of CYP2D6 inhibitors, differences in outcome definitions, and importantly, ethnic variation in allele frequency [45,46]. Consequently, the clinical utility of *CYP2D6* genotyping has been debated. To address this, a recent meta-analysis with bias-adjusted analysis of 33 studies in Caucasian and Asian populations helped reduce between-study heterogeneity [47]. The analysis confirmed that individuals with PM phenotypes had an increased risk of breast cancer recurrence and/or mortality associated with reduced CYP2D6 activity, while IM showed modest effects in European populations but larger, likely biased, effects in Asian cohorts. However, differences in allele prevalence between populations may limit the generalizability of the findings [47].

On the other hand, population pharmacokinetic models suggest that patients with impaired CYP2D6 metabolism might require higher tamoxifen doses (40 mg/day for IMs and 80 mg/day for PMs) to achieve therapeutic endoxifen levels comparable to those of normal metabolizers (NMs) on the standard 20 mg/day dose [48]. Prospective studies support this approach, showing that genotype-guided dose escalation can normalize endoxifen concentrations in IMs, although increases in PMs are more limited and may involve alternative metabolic pathways [49,50]. On this basis, some clinical guidelines propose either increasing tamoxifen doses or switching to aromatase inhibitors (AIs) in patients predicted to have low CYP2D6 activity [11,51].

Nevertheless, the TARGET-1 trial found that, despite achieving higher endoxifen exposure in patients with impaired metabolism, there was no improvement in progression-free survival (PFS) at 6 months [52]. These results point out the need for validation in larger cohorts with longer follow-up to confirm or refute the clinical benefit of genotype-guided dosing. Conversely, in patients who experience unacceptable side effects, low-dose tamoxifen has been shown to provide a more favorable toxicity profile compared to the standard dose, with strong indirect evidence supporting its antitumor efficacy [53].

Taken together, these findings suggest that tamoxifen dosing may be tailored to individual patient characteristics, whether to overcome reduced metabolism or to mitigate toxicity. However, further large-scale and long-term studies are required to determine whether such strategies translate into improved clinical outcomes.

Another important enzyme in tamoxifen metabolism is CYP3A5, along with its paralog, CYP3A4. *CYP3A5* has several genetic variants that categorize individuals as expressor or nonexpressor [54]. The most common variant, CYP3A5*3, is prevalent in many populations and results in null enzyme function, which may lead to lower concentrations of endoxifen and DM-TAM [55]. Despite this, a study by Wegman et al. reported that postmenopausal patients homozygous for the CYP3A5*3 allele exhibited improved recurrence-free survival (RFS) in a tamoxifen-treated cohort, which is an unexpected finding considering the reduced metabolic capacity associated with this allele [56]. In another cohort including Caucasian and African American patients, interethnic variability was apparent, with CYP3A5*3 more common in Caucasians and CYP3A5*6 more frequent in African Americans. CYP3A5*3 did not significantly affect steady-state tamoxifen or metabolite levels, likely due to compensatory activity from other CYP enzymes. However, carriers of at least one CYP3A5*6 allele were more likely to present with larger tumors and more advanced disease, suggesting a possible role in tumor progression or linkage with other genes, although further investigation is warranted given the allele’s rarity [57].

Similarly, CYP3A4, the primary isozyme of the CYP3A subfamily, has significant genetic variation as well, such as the CYP3A4*22 allele, which reduces the expression of the enzyme [58]. Interestingly, CYP3A4*22 carriers tend to achieve higher concentrations of tamoxifen and its metabolites, likely due to decreased intestinal CYP3A4 activity and reduced first-pass metabolism, resulting in increased systemic [15,59,60,61].

Beyond CYP2D6 and CYP3A enzymes, CYP2C19, CYP2C8/9, and CYP2B6 play minor roles in tamoxifen metabolism but can still significantly influence treatment metabolism.

For instance, *CYP2C19* polymorphisms affect norendoxifen (NorEND) levels, a tertiary metabolite of tamoxifen with potent aromatase-inhibitory properties, formed via N-demethylation of endoxifen. CYP2C19*2, a loss-of-function allele, is linked to lower NorEND levels, potentially impacting tamoxifen’s overall efficacy by affecting the peripheral conversion of androgens to estrogen [62]. However, some studies suggest that patients with the CYP2C19*2 heterozygous or homozygous genotypes may experience longer time-to-treatment failure and better overall breast cancer survival rates compared to those with the wild-type allele [63,64].

In contrast, the CYP2C19*17 allele, associated with ultra-rapid metabolism, leads to higher 4-OH-TAM levels, correlating with better outcomes and reduced breast cancer recurrence [65]. Additionally, the CYP2C19*17 variant has been linked to a reduced risk of breast cancer, as it also metabolizes endogenous estrogen, potentially lowering its levels and impacting cancer progression [66]. A meta-analysis has confirmed the association between the CYP2C19*2 and *17 genotypes with improved survival rates in breast cancer patients treated with tamoxifen [67]. However, inconsistencies in the literature persist [68,69], and to date, no formal clinical guidelines or recommendations exist for adjusting tamoxifen therapy based on *CYP2C19* genotype.

Likewise, *CYP2C9*, a highly polymorphic gene with CYP2C9*2 and CYP2C9*3 alleles, is associated with reduced enzyme activity and lower steady-state concentrations of active metabolites [7,70]. However, no definitive study has linked *CYP2C9* variants to survival outcomes [41].

Closely related to CYP2C9, CYP2C8 also contributes to tamoxifen metabolism, particularly in the formation of 4-OH-TAM. Notably, CYP2C8*3, a reduced-function allele, has been associated with altered pharmacokinetics in tamoxifen-treated patients and early breast cancer-related events [71]. This association implies that *CYP2C8* polymorphisms could influence relapse risk, but further large-scale studies are needed to clarify their clinical significance.

Notably, the CYP2C9*2 and CYP2C8*3 alleles are often found to co-segregate within families, suggesting a potential shared genetic background. The proximity of these alleles raises the possibility of a long haplotype, with potential implications for pharmacogenetic studies in populations carrying both variants [72].

Finally, CYP2B6, though less involved, affects 4-OH-TAM levels [73]. Higher levels of 4-OH-TAM are generally associated with better treatment outcomes; however, certain genetic variations in *CYP2B6*, such as the SNP *rs3211371*, have been linked to poorer survival rates, particularly in premenopausal women [74].

Table 1 summarizes the different gene variations, their impact on metabolism and outcomes, and proposed clinical recommendations.

### 3.3. Drug-Phase II Metabolizing Enzyme Interactions

SULT enzymes are phase II liver enzymes that play a critical role in detoxifying both endogenous and xenobiotic compounds, including tamoxifen. These enzymes catalyze the transfer of a sulfonyl group to nucleophilic groups on substrates, increasing their solubility and facilitating their excretion.

Research indicates that low activity of SULT1A1, attributed to specific SNPs in the 3′-untranslated region (UTR) of the *SULT1A1* gene, such as *rs6839* and *rs1042157*, is associated with higher concentrations of endoxifen and 4-OH-TAM compared to individuals with medium or high enzyme activity [86]. This suggests that decreased SULT1A1 activity could theoretically enhance the efficacy of tamoxifen treatment by increasing the availability of these metabolites. However, despite the expected efficacy enhancement, the SULT1A1*2 allele, which correlates with lower SULT1A1 activity, has also been linked to an increased hazard of death [75], and approximately three times the risk of death compared to individuals with the common allele homozygosity or heterozygosity [76]. Furthermore, a meta-analysis suggests that the reduced function SNP *rs9282861* of *SULT1A1* may be associated with breast cancer risk, particularly among Asian populations, but not in Caucasians [87]. The discrepancy between phenotype and predicted outcomes could be attributed to the sulfation of 4-OH-TAM, which may alter its pharmacokinetics or modify its receptor-binding properties, potentially affecting tamoxifen’s therapeutic efficacy. Moreover, tamoxifen treatment may stimulate the sulfation of other substrates, leading to unwanted effects [88]. Interestingly, a descriptive study reported that carriers of alleles associated with reduced SULT1A2 enzyme activity (SULT1A2*2 and SULT1A2*3) exhibit higher levels of 4-OH-TAM and endoxifen. Notably, the presence of just one defective *SULT1A2* allele appears sufficient to slow down the inactivation of these metabolites, thereby helping to maintain optimal levels of 4-OH-TAM and endoxifen [77].

Approximately 75% of the tamoxifen dose is excreted into the biliary system as glucuronides through the action of UGTs. This process is essential in maintaining optimal levels of both 4-OH-TAM and endoxifen. Variations in glucuronidation patterns could impact the half-lives of circulating tamoxifen and its metabolites, potentially influencing their effectiveness [89,90].

Variants in UGT2B15, a key enzyme involved in the metabolism of steroid substrates and exogenous compounds such as 4-OH-TAM, have been associated with altered tamoxifen pharmacokinetics. Individuals carrying the C allele of *UGT2B15 c.1568A > C* (rs4148269) tend to exhibit lower plasma concentrations of tamoxifen compared with those harboring the wild-type genotype [91]. Regarding clinical outcomes, this variant may influence overall survival and recurrence in tamoxifen-treated patients, although further validation in more homogeneous cohorts with comparable cancer characteristics is required [75]. In addition, the UGT2B15*2 variant (rs1902023) has been linked to a reduced risk of breast cancer relapse, suggesting a potentially protective role during tamoxifen therapy [78]. By contrast, UGT1A4, another member of the UGT family, can directly glucuronidate tamoxifen. Patients carrying the *UGT1A4* (rs869283) G/A or A/A genotypes derived less benefit from adjuvant tamoxifen treatment compared to those with the G/G genotype, suggesting a potential prognostic impact [92].

### 3.4. Drug Transporter Interactions

Drug transporters, including uptake and efflux proteins, are critical determinants of drug disposition and response. These transporters regulate the transmembrane movement of drugs and their metabolites. They are broadly classified into two main families: efflux transporters of the ATP-binding cassette (ABC) family and uptake transporters of the solute carrier (SLC) family. Genetic polymorphisms in transporter genes have been shown to significantly impact the expression, subcellular localization, substrate specificity, and intrinsic transport activity of these proteins, ultimately affecting the bioavailability of drug substrates [93].

In the context of tamoxifen, the active metabolites endoxifen and 4-OH-TAM are substrates of P-glycoprotein (P-gp), an efflux transporter encoded by the *ABCB1* gene that acts as a barrier, limiting the intracellular accumulation of these active metabolites in target tissues [94,95]. Variants in the ABCB1 gene, particularly the *ABCB1* 3435C > T (rs1045642) polymorphism, play an important role in modulating P-gp expression and function. For instance, P-gp protein levels were reported to be approximately twofold higher in individuals carrying the homozygous CC genotype compared to the TT genotype [79], suggesting that this variant may lead to reduced tamoxifen and metabolite bioavailability through enhanced efflux activity [80]. Consistently, patients with the homozygous TT genotype exhibit lower ABCB1 expression, correlating with diminished P-gp function relative to CC carriers [80]. Clinically, these functional differences have been associated with prognosis: patients with the heterozygous ABCB1 3435 CT genotype showed significantly shorter disease-free survival (DFS) compared to those with the homozygous 3435 CC genotype, with risk of recurrence increased by five times, whereas carriers of the TT genotype showed no difference [81]. Nevertheless, large prospective studies are required to confirm the clinical impact of ABCB1 polymorphisms on tamoxifen efficacy.

Additionally, members of the SLC family, particularly organic anion-transporting polypeptides (OATPs) encoded by the *SLCO* gene family, are implicated in the transport of tamoxifen and its metabolites. The *SLCO1B1 T521C* polymorphism has been associated with poor overall survival (OS), with patients carrying the wild-type *T/T* genotype exhibiting higher OS rates compared to homozygous variant *C/C* or heterozygous *C/T* genotypes [82]. This SNP is also associated with lower intracellular levels of tamoxifen and endoxifen [83,84]. *SLCO2B1 c.935G > A* polymorphism, on the other hand, is associated with higher endoxifen concentrations, with tamoxifen levels significantly elevated in carriers of the variant compared to wild-type individuals. Furthermore, *SLCO1A2 c.38A > G* has been linked to a reduction in ADRs [85].

Overall, the variability in tamoxifen metabolism and endoxifen plasma levels is shaped by the interplay of Phase I and II metabolic enzymes and drug transporters, each contributing to a different extent. Pharmacokinetic modeling studies have demonstrated that CYP2D6 activity is the dominant factor driving endoxifen concentrations, with minor contributions by other DMEs and external factors [96,97]. These state-of-the-art modeling approaches integrate pharmacogenetic and clinical information to more accurately predict endoxifen exposure and support personalized tamoxifen therapy.

## 4. Multigenic Variations Influence on Tamoxifen Therapy

The foundation of pharmacogenomics lies in the complex process of identifying genetic variations, understanding their functional implications, and ultimately linking them to drug response phenotypes. However, this approach is often complicated by the presence of multiple genetic variants within individuals, challenging the traditional analysis of how a single gene variant impacts drug response. Additionally, focusing solely on two factors (DDIs and DGIs) does not fully explain variations in serum drug levels. Mismatches between expected and actual drug metabolism capacities can arise from genetic changes in alternative metabolic pathways. This suggests the potential importance of adopting a panel-based approach, rather than focusing solely on a single gene, to better understand variations in metabolism [98,99].

For example, in the case of reduced CYP2D6 activity, it is conventionally expected that metabolite levels would be low. However, in an intriguing study observation, patients with both CYP3A4 PM and CYP2D6 PM phenotypes exhibit higher median plasma levels of tamoxifen and its metabolites, comparable to those of CYP2D6 NMs [100]. This suggests a potential compensatory effect by CYP3A4 PM, which could offset the reduction in endoxifen concentrations typically associated with CYP2D6 inactivity, particularly due to elevated 4-OH-TAM concentrations. Similarly, treatment outcomes can differ based on combined genetic variations.

Furthermore, patients with *CYP2D6* PM and *SULT1A1* NM genotypes show a significant 62% decrease in the risk of distant recurrence with tamoxifen compared to those with *CYP2D6* NM genotypes and carriers of the *SULT1A1* PM allele [101]. Interestingly, when considering *CYP2D6* and *SULT1A2* together, *CYP2D6* NMs with *SULT1A2* PM alleles maintain optimal plasma levels of endoxifen and 4-OH-TAM [77]. Furthermore, the combination of CYP2D6 NM and CYP2C19 UM status may confer longer relapse-free survival and disease-free survival in patients [65]. Conversely, individuals with low CYP2D6 activity and high CYP2C19 metabolism may experience shorter recurrence-free survival and breast cancer-specific survival, particularly among premenopausal women. This could be due to the development of hormone-independent tumours with more aggressive biology. For these individuals, decreased CYP2D6 activity, in combination with a more aggressive tumour type, may lead to worse clinical outcomes [102].

In addition to the aforementioned gene interactions, it is crucial to consider the combined effects of DMEs and transporters on treatment outcomes. For example, patients with *CYP2D6* IM and the homozygous *CC* genotype of *ABCB1 C3435T* were found to have significantly shorter times to recurrence compared to when these genetic factors are considered separately [16,103]. Similarly, the combination of *ABCC2 −24C > T* and *ABCB1 3435C > T* genotypes has been associated with an increased risk of disease recurrence, particularly bone metastasis in Thai population [104].

Table 2 summarizes the cumulative effects of multiple gene variations on tamoxifen metabolism and outcomes, along with their clinical implications.

## 5. Pharmacogenetic and Drug–Drug Interactions Effects on Tamoxifen Therapy

Various polymorphisms in DMEs contribute to inter-individual variations in plasma concentrations of tamoxifen and its metabolites. However, the complexity of tamoxifen metabolism extends beyond genetic polymorphisms alone. Serial measurements of tamoxifen and its metabolites in plasma have revealed intra-individual variations, even among patients with similar genotype groups [73]. One of the potential contributing factors is comedication. The increased likelihood of DDIs is a risk that is further magnified when genetic factors are considered. This interplay gives rise to a specific subset of pharmacogenetic interactions known as DDGIs, where a DDI occurs on top of a DGI. Notably, a large retrospective study of over 36,000 participants showed that patients with DDGIs/DGGIs represented 5.9% of the total cohort and 14.7% of those with an actionable pharmacogenetic interaction, pointing to the clinical significance of these combined interactions [105].

Depression affects approximately 30.2% of breast cancer patients, reaching as high as 83% in certain populations. Frontline therapies for managing depression and anxiety in these patients include serotonin reuptake inhibitors (SSRIs) and serotonin and norepinephrine reuptake inhibitors (SNRIs) [106]. However, SSRIs and SNRIs can inhibit CYP2D6. This inhibition can result in phenoconversion, where a discrepancy exists between genotype-based predictions of drug metabolism and an individual’s actual metabolic capacity [107]. This phenomenon was demonstrated in a study by Mostafa et al., who observed an increase in actionable genotypes due to phenoconversion in a significant proportion of patients. Specifically, the number of *CYP2D6* PMs increased from 5.4% (predicted by genotype) to 24.7% (adjusted phenotype) through phenoconversion [108].

Moreover, the efficacy and potential side effects of these medications can fluctuate depending on an individual’s *CYP2D6* genotype and the potency of the inhibitor [109]. In a prospective trial, concurrent use of CYP2D6 inhibitors with varying strengths significantly reduced mean plasma endoxifen concentrations, with the extent of reduction directly corresponding to the inhibitor’s potency. For instance, except for patients with CYP2D6 UMs, those administered strong CYP2D6 inhibitors were phenotypically converted to PMs. Furthermore, UM patients treated with weak or potent CYP2D6 inhibitors displayed lower endoxifen plasma levels, resembling those seen in patients with NM or IM phenotypes, emphasizing the significance of both genotype and inhibitor potency in phenoconversion [110]. Similarly, a study that incorporated tamoxifen and endoxifen concentrations alongside genetic data across six reported studies revealed that during concurrent use of a potent CYP2D6 inhibitor, endoxifen production was significantly inhibited by 65%. Consequently, genetically predicted NMs displayed endoxifen formation levels similar to those of CYP2D6 PMs [111]. In confirmation, switching from strong to weak inhibitors was deemed safe and feasible, resulting in clinically relevant rises in endoxifen concentrations without causing psychiatric problems or antidepressant-related adverse effects [112].

Taken together, accurate assessment of CYP2D6 phenotypes from genotype alone is insufficient for patients taking a CYP2D6 substrate alongside enzyme inhibitors. Consequently, the activity score should be adjusted by multiplying the score by zero for strong inhibitors and by 0.5 for moderate inhibitors [113]. Recommendations now advise against concomitant use of moderate and strong CYP2D6-inhibiting drugs [11].

In addition to CYP2D6, CYP3A enzymes play a crucial role in tamoxifen metabolism, and their inhibition or induction can affect treatment outcomes. Notably, Stefan et al. explored the potential to manipulate metabolic enzyme inducers and inhibitors to increase systemic endoxifen exposure, particularly for patients with CYP2D6 PM or inadequate endoxifen concentrations despite tamoxifen doses [114]. Their hypothesis was that elevating endoxifen concentrations could be achieved by inducing its formation and inhibiting its breakdown through co-administration of probenecid, a CYP3A4 inducer and pan-UGT inhibitor. Indeed, probenecid led to a clinically relevant increase of endoxifen concentrations by 24%, alongside a 110% rise in the metabolic ratio of endoxifen to tamoxifen in patients receiving both tamoxifen and probenecid compared to tamoxifen monotherapy [114].

However, this hypothesis does not always hold when applied to other medications. In a case study, a breast cancer patient with CYP2D6 IM experienced a potent interaction between tamoxifen and rifampin, a CYP3A inducer and antibiotic. Measuring tamoxifen and metabolite levels before and after rifampin administration clearly demonstrated a reduction in metabolite formation, with endoxifen concentrations dropping from >40 nM to 15.8 nM, which was restored once rifampin was discontinued [115]. Likewise, integrated data from breast cancer patients co-medicated with rifampicin and tamoxifen revealed substantial alterations in tamoxifen pharmacokinetics, resulting in endoxifen levels dropping below the therapeutic threshold [111]. Although enzyme induction would traditionally be expected to enhance endoxifen formation, the opposite effect is observed. This is likely due to simultaneous induction of phase II metabolism and drug transporters, leading to enhanced clearance of endoxifen and other intermediate metabolites of tamoxifen.

Further research on co-medication in HIV patients undergoing tamoxifen therapy revealed that antiretroviral therapy (ART), which contains efavirenz a CYP3A, CYP2B6, and the UGTs inducer, significantly influence the levels of DM-TAM and the metabolic ratios of tamoxifen to DM-TAM and DM-TAM to endoxifen, albeit without significant effects on endoxifen levels [116].

On the other hand, a study evaluating the impact of weak or moderate/potent CYP3A4 inhibitors found no significant impact on plasma endoxifen levels unless patients were classified according to CYP2D6 phenotype. Specifically, in CYP2D6 NMs, concurrent use of moderate or strong CYP3A4 inhibitors (e.g., amiodarone, clarithromycin, ciprofloxacin, diltiazem, fluconazole, and fusidic acid) led to markedly reduced endoxifen levels compared to patients not receiving these inhibitors. However, weak inhibitors showed no significant effect [117]. The study did not assess the impact of other CYP2D6 phenotypes and CYP3A4 inhibitors on plasma endoxifen levels due to insufficient patient numbers.

Table 3 summarizes the different drug–drug interactions and gene variation effects on tamoxifen metabolism and outcomes, along with the corresponding clinical recommendations.

## 6. Clinical Management of Tamoxifen Response Variability

### 6.1. Pharmacogenetic Testing

Pharmacogenetic testing in tamoxifen therapy focuses primarily on genotyping the *CYP2D6* gene. Determining a patient’s CYP2D6 metabolizer status can guide dose adjustments and help predict treatment efficacy and toxicity. Nevertheless, despite endorsement from multiple international guidelines [118,119], real-world integration of *CYP2D6* testing into clinical practice remains limited and inconsistent.

One of the main challenges stems from inconsistencies in phenotype classifications, allele interpretations, and dosing recommendations across consortia (such as CPIC, DPWG, and others) [120]. Furthermore, translating genotypes into actionable clinical recommendations is complicated by several practical barriers. Research-related barriers include the lack of strong outcome data linking CYP2D6 variants to tamoxifen response. Practical barriers involve the poor integration of pharmacogenetic data into electronic health records (EHRs) [121,122]. Awareness and knowledge barriers among clinicians are also critical: for example, one study reported that although 64% of healthcare providers had some familiarity with pharmacogenetics, the actual implementation of PGx testing in clinical practice remained low. The main challenges cited by providers were limited access to testing and insufficient knowledge on test interpretation [123]. Another study further highlighted that the lack of formal training and limited expertise in pharmacogenetics continues to undermine the clarity and confidence required for effective clinical decision-making [124]. Despite these obstacles, pharmacogenetic testing has consistently been shown to be cost-effective and even cost-saving [125]. Addressing these barriers through better training, improved access to testing, and integration of PGx information into clinical workflows will be essential to translating pharmacogenetic findings into routine practice.

Some healthcare institutions, such as the Mayo Clinic [126], have introduced *CYP2D6* genotyping into clinical workflows, demonstrating feasibility and benefits. In parallel, several initiatives such as the U-PGx project [127], the medeA model in Spain [128], the PROGRESS study in the UK [129], among others, are working to promote standardized pharmacogenomics implementation. Yet, only a few of these programs explicitly include *CYP2D6*-tamoxifen testing in their scope.

Meanwhile, some laboratories now offer pharmacogenetic passports, comprehensive multi-gene panels that provide a patient’s genetic profile across several pharmacogenes [130]. This genetic information equips clinicians to consider potential interactions and make more informed prescribing decisions.

Ultimately, while genotyping provides valuable insights into DME variations, it does not fully capture the dynamic nature of enzyme activity, which is influenced by environmental aspects, drug–drug interactions [131], disease state [132], inflammation [133], and other intrinsic and extrinsic factors, pointing out the need for complementary approaches such as therapeutic drug monitoring (TDM) or probe-based phenotyping.

### 6.2. Therapeutic Drug Monitoring of Endoxifen

TDM in tamoxifen therapy involves measuring plasma concentrations of endoxifen to guide individualized dose adjustments and ensure therapeutic efficacy. An endoxifen level of ≥15–16 nM has been associated with improved relapse-free survival; nonetheless, approximately 20–24% of patients on the standard 20 mg/day dose fail to achieve the threshold [32,134]. Despite this association, the large prospective CYPTAM study found no significant differences in relapse-free survival across several proposed cut-off values, challenging the clinical utility of a fixed threshold model [135]. It is important to note that only a single endoxifen measurement was taken, which may not fully capture intra-variability over time due to adherence or temporary drug interactions, and that censoring at therapy switch and limited follow-up could also affect the detection of associations with outcomes.

On the other hand, the TOTAM study demonstrated that stratification based on *CYP2D6* genotype revealed differences in mean endoxifen levels both before and after dose escalation. Following dose escalation, the predefined endoxifen target was reached in 100% of NMs, 79% of IMs, and only 36% of PMs [134]. These findings suggest that, to achieve therapeutic endoxifen concentrations early in treatment, it is advisable to anticipate *CYP2D6* genotype status early in the treatment course. Moreover, for patients with functional *CYP2D6* activity, at least one TDM measurement is recommended to ensure adequate drug exposure. The TOTAM study thus clearly supports the feasibility and clinical value of TDM in personalizing tamoxifen treatment, offering the potential to halve the proportion of patients with subtherapeutic endoxifen levels without increasing toxicity.

Beyond its clinical utility, TDM is considered cost-effective, improving quality-adjusted life years (QALYs), and remains a valuable tool in real-world settings [136]. Typically, TDM is performed after at least three to four months of therapy, once steady-state levels are achieved, whereas *CYP2D6* genotyping can be done pre-treatment. Genotyping thus serves as a proactive tool for identifying patients at risk of underexposure, who may require early or closer monitoring. When used together, pre-treatment genotyping and post-treatment TDM form a complementary, two-step precision medicine strategy that enhances treatment personalization and ensures tamoxifen efficacy.

### 6.3. Phenotyping CYPs Activities Using Validated Probe Drugs

To overcome genotyping limitations, the tamoxifen-to-endoxifen metabolic ratio serves as a clinically relevant metric, directly reflecting CYP2D6-mediated tamoxifen conversion [137]. However, it cannot be assessed before treatment initiation and requires at least three to four months of therapy, potentially delaying early intervention in poor metabolizers.

Alternatively, in vivo phenotyping using probe drugs provides a more direct and reliable approach for assessing real-time enzyme function. For CYP2D6, the dextromethorphan metabolic ratio test, which measures urinary or plasma dextromethorphan-to-dextrorphan levels, strongly correlates with tamoxifen metabolism [138,139]. For CYP3A4/5, midazolam clearance test, which evaluates the conversion of midazolam to hydroxymidazolam, is the gold standard. Additionally, cocktail phenotyping approaches, such as the Pittsburgh [140], Geneva [141], and Basel [142] cocktails, enable simultaneous evaluation of multiple CYP enzymes using low-dose probe substrates, reducing logistical challenges while offering a comprehensive metabolic profile.

Despite their accuracy, exogenous probe drugs are not without drawbacks. Even at micro-doses, potential side effects remain a concern. To mitigate these risks, pharmacometabolomic studies have identified endogenous biomarkers that correlate with CYP activity. For CYP2D6, solanidine has emerged as a promising biomarker for non-invasive enzyme monitoring [143]. This was further validated in a subsequent study, which confirmed that the M414-to-solanidine metabolic ratio is an excellent predictor of the CYP2D6 PM phenotype [144]. Similarly, for CYP3A4, 4β-hydroxycholesterol, a product of cholesterol hydroxylation, is among the most extensively studied endogenous biomarkers [145,146].

From a clinical perspective, integrating both genotyping and phenotyping provides the most comprehensive assessment of drug metabolism [139,141]. While genotyping is crucial at treatment initiation, given the stability of the genome, it is equally important to monitor enzyme activity through phenotyping, particularly in scenarios where polypharmacy or pluropathology may alter enzymatic function. Ultimately, a comprehensive strategy that combines genotyping and phenotyping is essential for optimizing individualized pharmacotherapy, minimizing ADRs and preventing treatment inefficacy.

## 7. Conclusions

This review explores the complex interplay between individual genetic profiles and drug–drug interactions in shaping drug metabolism, treatment response, and clinical outcomes. It highlights the well-established role of pharmacogenetics in tamoxifen metabolism, while also acknowledging the inconsistencies observed in its association with treatment efficacy.

Based on current literature, a comprehensive approach is recommended. Pharmacogenetic testing should be conducted prior to treatment to identify altered metabolizers who are at higher risk of suboptimal tamoxifen response or adverse effects. This allows clinicians to anticipate inter-individual variability and consider alternative dosing strategies or therapies when necessary. Complementing this with TDM or probe-based phenotyping during treatment allows for real-time assessment of drug exposure and metabolic activity, capturing influences beyond genotype alone. Together, this dual strategy can improve personalized therapy, optimize treatment efficacy, and minimize the risk of adverse outcomes in patients receiving tamoxifen.

Nevertheless, routine clinical implementation remains limited by gaps in prospective outcome data, heterogeneous study designs, population diversity, and numerous real-world barriers. Future well-designed prospective trials, though challenging, are required to clarify these associations, confirm clinical utility, and resolve ongoing uncertainties.

Importantly, underestimating the clinical relevance of pharmacogenetics may lead to missed opportunities for optimizing therapy. Therefore, an inclusive strategy that prioritizes the identification and support of poor metabolizer subgroups is essential to promote more equitable and patient-centred care.

## Figures and Tables

**Table 1:** Pharmacogenetic interactions and their clinical implications.

Gene	Variant (s)	Functional Effect	Effect on Metabolism	Clinical Outcome	Clinical Recommendations	References
*CYP2D6*	PM/IM alleles (*4, *3, *41, *10…)	Reduced activity	↓ Endoxifen levels	Higher recurrence risk, poorer survival	Consider higher tamoxifen dose or switch to aromatase inhibitor	[19,20,21,22,23,24,25]
	Copy number variations	Increased activity	↑ Endoxifen levels	Better efficacy, but more side effects	Monitor for toxicity	[26]
*CYP3A5*	*3	Reduced activity	↓ DM-TAM and endoxifen levels	Mixed evidence on survival	Limited clinical impact; more studies needed	[55,56]
*CYP3A4*	*22	Reduced activity	↓ First-pass metabolism; ↑ Tamoxifen/metabolites levels	Potentially improved efficacy; less ADRs	No routine action; consider in polygenic context	[15,59,60,61]
*CYP2C19*	*2	Reduced activity	↓ NorEND levels	Conflicting results	More evidence needed before clinical use	[62,63,64,67]
	*17	Increased activity	↑ 4-OH-TAM levels	Lower risk for relapse	Consider as potential protective variant	[65,66,67]
*CYP2C8/9*	*2, *3	Reduced activity	↓ Endoxifen levels	Mixed evidence on survival	Insufficient evidence for clinical action	[7,70,71,72]
*SULT1A1*	*2	Reduced activity	↑ Endoxifen and 4-OH-TAM levels	Higher relapse risk and mortality	Potential predictor of tamoxifen response	[75,76]
*SULT1A2*	*2, *3	Reduced activity	↑ Endoxifen and 4-OH-TAM levels	No clinical studies	May help maintain optimal metabolite levels; needs validation	[77]
*UGT2B15*	*2	Reduced activity	↓ Tamoxifen plasma levels	Lower relapse risk	Potential prognostic marker; may act as protective variant	[78]
*ABCB1*	C3435T	Reduced activity	↓ Endoxifen intracellular concentration	Worse survival with CC/CT genotype	Variant allele carriers may respond better	[79,80,81]
*SLCO1B1*	T521C	Reduced activity	↓ Endoxifen in target tissue	Worse outcomes	Consider in multigene models	[82,83,84]
*SlCO1A2*	c.935G > A	Reduced activity	↑ Endoxifen	Reduction in ADRs	Potential predictor of tamoxifen ADRs	[85]

Arrows denote direction of change: ↓ reduction, ↑ elevation.

**Table 2:** Combined impact of multiple gene variations on tamoxifen metabolism and their clinical implications.

Variant A	Variant B	Impact on Metabolism and/or Outcome	Clinical Implication	References
CYP2D6 PM	CYP3A4 PM	↑ active metabolites levels compared to CYP2D6 NM	Potential compensatory effect via CYP3A4	[100]
	SULT1A1 NM	↑ risk of disease recurrence compared to SULT1A1 PM	Possible adverse impact on treatment outcomes	[101]
	CYP2C19 UM	↓ recurrence-free survival and breast cancer-specific survival	Potentially linked to more aggressive tumors; validation required	[102]
CYP2D6 IM	ABCB1 C3435T	↑ risk of disease recurrence	Potential risk factor for treatment failure	[16,103]
CYP2D6 NM	SULT1A2 PM	Maintain optimal endoxifen and OH-TAM levels	Potential protective effect	[77]
	CYP2C19 UM	↑ relapse-free survival and disease-free survival	Potential protective effect	[65]
ABCC2 −24C > T	ABCB1 C3435T	↑ risk of disease recurrence	May identify patients at higher recurrence risk	[104]

Arrows denote direction of change: ↓ reduction, ↑ elevation.

**Table 3:** DDGIs, their impact on tamoxifen metabolism, and related clinical recommendations.

DDIs	Effect on DMEs	Effect on Metabolism	Recommendations	References
Strong SSRIs	Strong CYP2D6 inhibition	↓ Endoxifen levels; phenoconversion: UM → PM and NM → PM	Avoid strong/moderate inhibitors; consider switching to AIs	[11,110,111,112,113]
Weak SSRIs	Weak CYP2D6 inhibition	↓ Endoxifen levels; phenoconversion: UM → NM/IM	No specific recommendations	
probenecid	CYP3A4 Inducer and pan-UGT inhibitor	↑ Endoxifen levels; ↑ Endoxifen/tamoxifen ratio	If validated, may serve as corrective therapy for CYP2D6 PMs	[114]
rifampin	CYP3A inducer	↓ Endoxifen levels	Potential DDIs; monitor endoxifen if co-administration unavoidable	[111,115]
Antiretroviral therapy (efavirenz)	CYP3A, CYP2B6 and UGT inducer	↓ DM-TAM; ↑ DM/TAM and ↑ DM/endoxifen ratios	Requires validation in larger cohorts	[116]
amiodarone, clarithromycin, ciprofloxacin, diltiazem, fluconazole, and fusidic acid	Strong CYP3A inhibitors and UGT inducer	↓ Endoxifen levels	Requires validation in larger cohorts	[117]

Arrows denote direction of change: ↓ reduction, ↑ elevation.

## Data Availability

Not applicable.

## Abbreviations

The following abbreviations are used in this manuscript:

4-Oh-tam	4-Hydroxy-Tamoxifen
ABC	ATP-Binding Cassette transporters
ADR	Adverse Drug Reaction
AIs	Aromatase Inhibitors
ART	Antiretroviral Therapy
CYP	Cytochrome P450 enzymes
DDIs	Drug–Drug Interactions
DDGIs	Drug–Drug–Gene Interactions
DGGIs	Drug–Gene–Gene Interactions
DGIs	Drug–Gene Interactions
DM-TAM	Desmethyl-Tamoxifen
DMEs	Drug Metabolism Enzymes
EHR	Electronic Health Records
ER+	Estrogen Receptor–Positive
GWAS	Genome-Wide Association Studies
IM	Intermediate Metabolizer
NM	Normal Metabolizer
NorEND	N-desmethyl-4-hydroxy-tamoxifen
OATPs	Organic Anion Transporting Polypeptides
P-gp	P-glycoprotein
PM	Poor Metabolizer
SLC	Solute Carrier transporters
SNRIs	Serotonin–Norepinephrine Reuptake Inhibitors
SSRIs	Selective Serotonin Reuptake Inhibitors
SULT	Sulfotransferases
TDM	Therapeutic Drug Monitoring
UGTs	UDP-glucuronosyltransferases
UM	Ultrarapid Metabolizer

## References

[1] Giaquinto A.N., Sung H., Miller K.D., Kramer J.L., Newman L.A., Minihan A., Jemal A., Siegel R.L. (2022). Breast Cancer Statistics, 2022. CA Cancer J. Clin..

[2] Jayasekera J., Zhao A., Schechter C., Lowry K., Yeh J.M., Schwartz M.D., O’Neill S., Wernli K.J., Stout N., Mandelblatt J. (2023). Reassessing the Benefits and Harms of Risk-Reducing Medication Considering the Persistent Risk of Breast Cancer Mortality in Estrogen Receptor–Positive Breast Cancer. J. Clin. Oncol..

[3] Eggemann H., Brucker C., Schrauder M., Thill M., Flock F., Reinisch M., Costa S.-D., Ignatov A. (2020). Survival Benefit of Tamoxifen in Male Breast Cancer: Prospective Cohort Analysis. Br. J. Cancer.

[4] Early Breast Cancer Trialists’ Collaborative Group (2005). Effects of Chemotherapy and Hormonal Therapy for Early Breast Cancer on Recurrence and 15-Year Survival: An Overview of the Randomised Trials. Lancet.

[5] Hahn M., Roll S.C. (2021). The Influence of Pharmacogenetics on the Clinical Relevance of Pharmacokinetic Drug–Drug Interactions: Drug–Gene, Drug–Gene–Gene and Drug–Drug–Gene Interactions. Pharmaceuticals.

[6] Jeiziner C., Stäuble C.K., Lampert M.L., Hersberger K.E., Meyer Z., Schwabedissen H.E. (2021). Enriching Medication Review with a Pharmacogenetic Profile—A Case of Tamoxifen Adverse Drug Reactions. Pharmgenom. Pers. Med..

[7] Mürdter T.E., Schroth W., Bacchus-Gerybadze L., Winter S., Heinkele G., Simon W., Fasching P.A., Fehm T., Eichelbaum M., German Tamoxifen and AI Clinicians Group (2011). Activity Levels of Tamoxifen Metabolites at the Estrogen Receptor and the Impact of Genetic Polymorphisms of Phase I and II Enzymes on Their Concentration Levels in Plasma. Clin. Pharmacol. Ther..

[8] Hodel F., De Min M.B., Thorball C.W., Redin C., Vollenweider P., Girardin F., Fellay J. (2024). Prevalence of Actionable Pharmacogenetic Variants and High-risk Drug Prescriptions: A Swiss Hospital-based Cohort Study. Clin. Transl. Sci..

[9] Nunez-Torres R., Pita G., Peña-Chilet M., López-López D., Zamora J., Roldán G., Herráez B., Álvarez N., Alonso M.R., Dopazo J. (2023). A Comprehensive Analysis of 21 Actionable Pharmacogenes in the Spanish Population: From Genetic Characterisation to Clinical Impact. Pharmaceutics.

[10] Jithesh P.V., Abuhaliqa M., Syed N., Ahmed I., El Anbari M., Bastaki K., Sherif S., Umlai U.-K., Jan Z., Gandhi G. (2022). A Population Study of Clinically Actionable Genetic Variation Affecting Drug Response from the Middle East. Npj Genom. Med..

[11] Goetz M.P., Sangkuhl K., Guchelaar H.J., Schwab M., Province M., Whirl-Carrillo M., Symmans W.F., McLeod H.L., Ratain M.J., Zembutsu H. (2018). Clinical Pharmacogenetics Implementation Consortium (CPIC) Guideline for CYP2D6 and Tamoxifen Therapy. Clin. Pharmacol. Ther..

[12] Helland T., Henne N., Bifulco E., Naume B., Borgen E., Kristensen V.N., Kvaløy J.T., Lash T.L., Alnæs G.I.G., van Schaik R.H. (2017). Serum concentrations of active tamoxifen metabolites predict long-term survival in adjuvantly treated breast cancer patients. Breast Cancer Res..

[13] Hammarström M., Gabrielson M., Bergqvist J., Lundholm C., Crippa A., Bäcklund M., Wengström Y., Borgquist S., Eliasson E., Eriksson M. (2025). Influence of endoxifen on mammographic density: Results from the KARISMA-Tam trial. J. Natl. Cancer Inst..

[14] Tamoxifen Pathway, Pharmacokinetics. https://www.pharmgkb.org/pathway/PA145011119.

[15] Chen Y., Marcath L.A., Eliassen F.M., Lende T.H., Soiland H., Mellgren G., Helland T., Hertz D.L. (2021). Effect of Genetic Variability in 20 Pharmacogenes on Concentrations of Tamoxifen and Its Metabolites. J. Pers. Med..

[16] Powers J.L., Buys S.S., Fletcher D., Melis R., Johnson-Davis K.L., Lyon E., Malmberg E.M., McMillin G.A. (2016). Multigene and Drug Interaction Approach for Tamoxifen Metabolite Patterns Reveals Possible Involvement of CYP2C9, CYP2C19, and ABCB1. J. Clin. Pharmacol..

[17] Gaedigk A., Sangkuhl K., Whirl-Carrillo M., Twist G.P., Klein T.E., Miller N.A. (2019). The Evolution of PharmVar. Clin. Pharmacol. Ther..

[18] Gaedigk A., Simon S., Pearce R., Bradford L., Kennedy M., Leeder J. (2008). The CYP2D6 Activity Score: Translating Genotype Information into a Qualitative Measure of Phenotype. Clin. Pharmacol. Ther..

[19] Saladores P., Mürdter T., Eccles D., Chowbay B., Zgheib N.K., Winter S., Ganchev B., Eccles B., Gerty S., Tfayli A. (2015). Tamoxifen Metabolism Predicts Drug Concentrations and Outcome in Premenopausal Patients with Early Breast Cancer. Pharmacogenom. J..

[20] Li X., Li Z., Li L., Liu T., Qian C., Ren Y., Li Z., Chen K., Ji D., Zhang M. (2024). Toremifene, an Alternative Adjuvant Endocrine Therapy, Is Better Than Tamoxifen in Breast Cancer Patients with CYP2D6*10 Mutant Genotypes. Cancer Res. Treat..

[21] Goetz M.P., Suman V.J., Hoskin T.L., Gnant M., Filipits M., Safgren S.L., Kuffel M., Jakesz R., Rudas M., Greil R. (2013). CYP2D6 Metabolism and Patient Outcome in the Austrian Breast and Colorectal Cancer Study Group Trial (ABCSG) 8. Clin. Cancer Res..

[22] Karle J., Bolbrinker J., Vogl S., Kreutz R., Denkert C., Eucker J., Wischnewsky M., Possinger K., Regierer A.C. (2013). Influence of CYP2D6-Genotype on Tamoxifen Efficacy in Advanced Breast Cancer. Breast Cancer Res. Treat..

[23] Lammers L.A., Mathijssen R.H.J., van Gelder T., Bijl M.J., de Graan A.-J.M., Seynaeve C., van Fessem M.A., Berns E.M., Vulto A.G., van Schaik R.H.N. (2010). The Impact of CYP2D6-Predicted Phenotype on Tamoxifen Treatment Outcome in Patients with Metastatic Breast Cancer. Br. J. Cancer.

[24] Malash I., Mansour O., Shaarawy S., Abdellateif M.S., Omar A., Gaafar R., Zekri A.-R.N., Ahmed O.S., Bahnassy A. (2020). The Role of CYP2D6 Polymorphisms in Determining Response to Tamoxifen in Metastatic Breast Cancer Patients: Review and Egyptian Experience. Asian Pac. J. Cancer Prev. APJCP.

[25] Newman W.G., Hadfield K.D., Latif A., Roberts S.A., Shenton A., McHague C., Lalloo F., Howell S., Evans D.G. (2008). Impaired Tamoxifen Metabolism Reduces Survival in Familial Breast Cancer Patients. Clin. Cancer Res..

[26] He W., Eriksson M., Eliasson E., Grassmann F., Bäcklund M., Gabrielson M., Hammarström M., Margolin S., Thorén L., Wengström Y. (2021). CYP2D6 Genotype Predicts Tamoxifen Discontinuation and Drug Response: A Secondary Analysis of the KARISMA Trial. Ann. Oncol..

[27] Henry N.L., Rae J.M., Li L., Azzouz F., Skaar T.C., Desta Z., Sikora M.J., Philips S., Nguyen A., Storniolo A.M. (2009). Association between CYP2D6 Genotype and Tamoxifen-Induced Hot Flashes in a Prospective Cohort. Breast Cancer Res. Treat..

[28] Ramón y Cajal T., Altés A., Paré L., del Rio E., Alonso C., Barnadas A., Baiget M. (2010). Impact of CYP2D6 Polymorphisms in Tamoxifen Adjuvant Breast Cancer Treatment. Breast Cancer Res. Treat..

[29] Wickramage I., Tennekoon K.H., Ariyaratne M.A.Y., Hewage A.S., Sundralingam T. (2017). CYP2D6 Polymorphisms May Predict Occurrence of Adverse Effects to Tamoxifen: A Preliminary Retrospective Study. Breast Cancer Targets Ther..

[30] Goetz M., Kamal A., Ames M. (2008). Tamoxifen Pharmacogenomics: The Role of CYP2D6 as a Predictor of Drug Response. Clin. Pharmacol. Ther..

[31] Hwang G.S., Bhat R., Crutchley R.D., Trivedi M.V. (2018). Impact of CYP2D6 Polymorphisms on Endoxifen Concentrations and Breast Cancer Outcomes. Pharmacogenom. J..

[32] Madlensky L., Natarajan L., Tchu S., Pu M., Mortimer J., Flatt S.W., Nikoloff D.M., Hillman G., Fontecha M.R., Lawrence H.J. (2011). Tamoxifen Metabolite Concentrations, CYP2D6 Genotype, and Breast Cancer Outcomes. Clin. Pharmacol. Ther..

[33] Braal C.L., Westenberg J.D., Buijs S.M., Abrams S., Mulder T.A.M., van Schaik R.H.N., Koolen S.L.W., Jager A., Mathijssen R.H.J. (2022). Factors Affecting Inter-Individual Variability in Endoxifen Concentrations in Patients with Breast Cancer: Results from the Prospective TOTAM Trial. Breast Cancer Res. Treat..

[34] Ximenez J.P.B., de Andrade J.M., Marques M.P., Coelho E.B., Suarez-Kurtz G., Lanchote V.L. (2019). Hormonal Status Affects Plasma Exposure of Tamoxifen and Its Main Metabolites in Tamoxifen-Treated Breast Cancer Patients. BMC Pharmacol. Toxicol..

[35] Sanchez-Spitman A.B., Böhringer S., Dezentjé V.O., Gelderblom H., Swen J.J., Guchelaar H.-J. (2024). A Genome-Wide Association Study of Endoxifen Serum Concentrations and Adjuvant Tamoxifen Efficacy in Early-Stage Breast Cancer Patients. Clin. Pharmacol. Ther..

[36] Khor C.C., Winter S., Sutiman N., Mürdter T.E., Chen S., Lim J.S.L., Li Z., Li J., Sim K.S., Ganchev B. (2023). Cross-Ancestry Genome-Wide Association Study Defines the Extended CYP2D6 Locus as the Principal Genetic Determinant of Endoxifen Plasma Concentrations. Clin. Pharmacol. Ther..

[37] Sanchez-Spitman A., Dezentjé V., Swen J., Moes D.J.A.R., Böhringer S., Batman E., van Druten E., Smorenburg C., van Bochove A., Zeillemaker A. (2019). Tamoxifen Pharmacogenetics and Metabolism: Results From the Prospective CYPTAM Study. J. Clin. Oncol..

[38] Stearns V., ONeill A., Schneider B.P., Skaar T.C., Liu M.C., Lohrisch C., Goetz M.P., Vallejos C.S., Sparano J.A., Villa D. (2025). CYP2D6 activity in patients with metastatic breast cancer treated with single agent tamoxifen: Results from ECOG-ACRIN E3108. Breast Cancer Res. Treat..

[39] Thorén L., Lindh J.D., Molden E., Kristiansen Kringen M., Bergh J., Eliasson E., Margolin S. (2025). CYP2D6 genotype and outcome in tamoxifen treated early breast cancer. Acta Oncol..

[40] Ahern T.P., Hertz D.L., Damkier P., Ejlertsen B., Hamilton-Dutoit S.J., Rae J.M., Regan M.M., Thompson A.M., Lash T.L., Cronin-Fenton D.P. (2017). Cytochrome P-450 2D6 (CYP2D6) Genotype and Breast Cancer Recurrence in Tamoxifen-Treated Patients: Evaluating the Importance of Loss of Heterozygosity. Am. J. Epidemiol..

[41] Dezentjé V.O., van Schaik R.H.N., Vletter-Bogaartz J.M., van der Straaten T., Wessels J.a.M., Kranenbarg E.M.-K., Berns E.M., Seynaeve C., Putter H., van de Velde C.J.H. (2013). CYP2D6 Genotype in Relation to Tamoxifen Efficacy in a Dutch Cohort of the Tamoxifen Exemestane Adjuvant Multinational (TEAM) Trial. Breast Cancer Res. Treat..

[42] Hertz D.L., Kidwell K.M., Hilsenbeck S.G., Oesterreich S., Osborne C.K., Philips S., Chenault C., Hartmaier R.J., Skaar T.C., Sikora M.J. (2017). CYP2D6 genotype is not associated with survival in breast cancer patients treated with tamoxifen: Results from a population-based study. Breast Cancer Res. Treat..

[43] Province M.A., Goetz M.P., Brauch H., Flockhart D.A., Hebert J.M., Whaley R., Suman V.J., Schroth W., Winter S., Zembutsu H. (2014). CYP2D6 genotype and adjuvant tamoxifen: Meta-analysis of heterogeneous study populations. Clin. Pharmacol. Ther..

[44] Goetz M.P., Suman V.J., Nakamura Y., Kiyotani K., Jordan V.C., Ingle J.N. (2019). Tamoxifen Metabolism and Breast Cancer Recurrence: A Question Unanswered by CYPTAM. J. Clin. Oncol..

[45] Baatjes K.J., Conradie M., Apffelstaedt J.P., Kotze M.J. (2017). Pharmacogenetics of Aromatase Inhibitors in Endocrine Responsive Breast Cancer: Lessons Learnt from Tamoxifen and CYP2D6 Genotyping. Anti-Cancer Agents Med. Chem. Former. Curr. Med. Chem. Anti-Cancer Agents.

[46] Turner A.J., Aggarwal P., Boone E.C., Haidar C.-E., Relling M.V., Derezinski A.D., Broeckel U., Gaedigk A. (2021). Identification of CYP2D6 Haplotypes That Interfere with Commonly Used Assays for Copy Number Variation Characterization. J. Mol. Diagn..

[47] MacLehose R.F., Ahern T.P., Collin L.J., Li A., Lash T.L. (2025). CYP2D6 Phenotype and Breast Cancer Outcomes: A Bias Analysis and Meta-Analysis. Cancer Epidemiol. Biomark. Prev..

[48] Puszkiel A., Arellano C., Vachoux C., Evrard A., Le Morvan V., Boyer J.-C., Robert J., Delmas C., Dalenc F., Debled M. (2021). Model-Based Quantification of Impact of Genetic Polymorphisms and Co-Medications on Pharmacokinetics of Tamoxifen and Six Metabolites in Breast Cancer. Clin. Pharmacol. Ther..

[49] Hertz D.L., Deal A., Ibrahim J.G., Walko C.M., Weck K.E., Anderson S., Magrinat G., Olajide O., Moore S., Raab R. (2016). Tamoxifen Dose Escalation in Patients with Diminished CYP2D6 Activity Normalizes Endoxifen Concentrations Without Increasing Toxicity. Oncologist.

[50] Khalaj Z., Baratieh Z., Nikpour P., Schwab M., Schaeffeler E., Mokarian F., Khanahmad H., Salehi R., Mürdter T.E., Salehi M. (2019). Clinical Trial: CYP2D6 Related Dose Escalation of Tamoxifen in Breast Cancer Patients with Iranian Ethnic Background Resulted in Increased Concentrations of Tamoxifen and Its Metabolites. Front. Pharmacol..

[51] Tamoxifen. https://www.clinpgx.org/chemical/PA451581/guidelineAnnotation/PA166104966.

[52] Tamura K., Imamura C.K., Takano T., Saji S., Yamanaka T., Yonemori K., Takahashi M., Tsurutani J., Nishimura R., Sato K. (2020). CYP2D6 Genotype–Guided Tamoxifen Dosing in Hormone Receptor–Positive Metastatic Breast Cancer (TARGET-1): A Randomized, Open-Label, Phase II Study. J. Clin. Oncol..

[53] Buijs S.M., Koolen S.L.W., Mathijssen R.H.J., Jager A. (2024). Tamoxifen Dose De-Escalation: An Effective Strategy for Reducing Adverse Effects?. Drugs.

[54] Gene-Specific Information Tables for CYP3A5. https://www.pharmgkb.org/page/cyp3a5RefMaterials.

[55] Zhang Y., Wang Z., Wang Y., Jin W., Zhang Z., Jin L., Qian J., Zheng L. (2024). CYP3A4 and CYP3A5: The Crucial Roles in Clinical Drug Metabolism and the Significant Implications of Genetic Polymorphisms. PeerJ.

[56] Wegman P., Elingarami S., Carstensen J., Stål O., Nordenskjöld B., Wingren S. (2007). Genetic Variants of CYP3A5, CYP2D6, SULT1A1, UGT2B15 and Tamoxifen Response in Postmenopausal Patients with Breast Cancer. Breast Cancer Res. BCR.

[57] Tucker A.N., Tkaczuk K.A., Lewis L.M., Tomic D., Lim C.K., Flaws J.A. (2005). Polymorphisms in Cytochrome P4503A5 (CYP3A5) May Be Associated with Race and Tumor Characteristics, but Not Metabolism and Side Effects of Tamoxifen in Breast Cancer Patients. Cancer Lett..

[58] Collins J.M., Wang D. (2021). Cytochrome P450 3A4 (CYP3A4) protein quantification using capillary western blot technology and total protein normalization. J. Pharmacol. Toxicol. Methods.

[59] Sanchez Spitman A.B., Moes D.J.A.R., Gelderblom H., Dezentje V.O., Swen J.J., Guchelaar H.J. (2017). Effect of CYP3A4*22, CYP3A5*3, and CYP3A Combined Genotypes on Tamoxifen Metabolism. Eur. J. Clin. Pharmacol..

[60] Teft W.A., Gong I.Y., Dingle B., Potvin K., Younus J., Vandenberg T.A., Brackstone M., Perera F.E., Choi Y.-H., Zou G. (2013). CYP3A4 and Seasonal Variation in Vitamin D Status in Addition to CYP2D6 Contribute to Therapeutic Endoxifen Level during Tamoxifen Therapy. Breast Cancer Res. Treat..

[61] Baxter S.D., Teft W.A., Choi Y.-H., Winquist E., Kim R.B. (2014). Tamoxifen-Associated Hot Flash Severity Is Inversely Correlated with Endoxifen Concentration and CYP3A4*22. Breast Cancer Res. Treat..

[62] Lim J.S.L., Sutiman N., Muerdter T.E., Singh O., Cheung Y.B., Ng R.C.H., Yap Y.S., Wong N.S., Ang P.C.S., Dent R. (2016). Association of CYP2C19*2 and Associated Haplotypes with Lower Norendoxifen Concentrations in Tamoxifen-treated Asian Breast Cancer Patients. Br. J. Clin. Pharmacol..

[63] Ruiter R., Bijl M.J., van Schaik R.H.N., Berns E.M.J.J., Hofman A., Coebergh J.-W.W., van Noord C., Visser L.E., Stricker B.H.C. (2010). CYP2C19*2 Polymorphism Is Associated with Increased Survival in Breast Cancer Patients Using Tamoxifen. Pharmacogenomics.

[64] van Schaik R.H.N., Kok M., Sweep F.C.G.J., van Vliet M., van Fessem M., Meijer-van Gelder M.E., Seynaeve C., Lindemans J., Wesseling J., Van ’t Veer L.J. (2011). The CYP2C19*2 Genotype Predicts Tamoxifen Treatment Outcome in Advanced Breast Cancer Patients. Pharmacogenomics.

[65] Schroth W., Antoniadou L., Fritz P., Schwab M., Muerdter T., Zanger U.M., Simon W., Eichelbaum M., Brauch H. (2007). Breast Cancer Treatment Outcome with Adjuvant Tamoxifen Relative to Patient CYP2D6 and CYP2C19 Genotypes. J. Clin. Oncol..

[66] Justenhoven C., Hamann U., Pierl C.B., Baisch C., Harth V., Rabstein S., Spickenheuer A., Pesch B., Brüning T., Winter S. (2009). CYP2C19*17 Is Associated with Decreased Breast Cancer Risk. Breast Cancer Res. Treat..

[67] Bai L., He J., He G.-H., He J.-C., Xu F., Xu G.-L. (2014). Association of CYP2C19 Polymorphisms with Survival of Breast Cancer Patients Using Tamoxifen: Results of a Meta- Analysis. Asian Pac. J. Cancer Prev. APJCP.

[68] Sanchez-Spitman A.B., Swen J.J., Dezentjé V.O., Moes D.J.A.R., Gelderblom H., Guchelaar H.J. (2021). Effect of CYP2C19 Genotypes on Tamoxifen Metabolism and Early-Breast Cancer Relapse. Sci. Rep..

[69] Mwinyi J., Vokinger K., Jetter A., Breitenstein U., Hiller C., Kullak-Ublick G.A., Trojan A. (2014). Impact of Variable CYP Genotypes on Breast Cancer Relapse in Patients Undergoing Adjuvant Tamoxifen Therapy. Cancer Chemother. Pharmacol..

[70] Marcath L., Deal A.M., Van Wieren E., Danko W., Walko C.M., Ibrahim J.G., Weck K.E., Jones D.R., Desta Z., McLeod H.L. (2017). Comprehensive Assessment of Cytochromes P450 and Transporter Genetics with Endoxifen Concentration during Tamoxifen Treatment. Pharmacogenet. Genom..

[71] Jernström H., Bågeman E., Rose C., Jönsson P.-E., Ingvar C. (2009). CYP2C8 and CYP2C9 Polymorphisms in Relation to Tumour Characteristics and Early Breast Cancer Related Events among 652 Breast Cancer Patients. Br. J. Cancer.

[72] Haeggström S., Ingelman-Sundberg M., Pääbo S., Zeberg H. (2022). The Clinically Relevant CYP2C8*3 and CYP2C9*2 Haplotype Is Inherited from Neandertals. Pharmacogenom. J..

[73] Woo H.I., Lee S.K., Kim J., Kim S.W., Yu J., Bae S.Y., Lee J.E., Nam S.J., Lee S.-Y. (2017). Variations in Plasma Concentrations of Tamoxifen Metabolites and the Effects of Genetic Polymorphisms on Tamoxifen Metabolism in Korean Patients with Breast Cancer. Oncotarget.

[74] Kuo S.-H., Yang S.-Y., You S.-L., Lien H.-C., Lin C.-H., Lin P.-H., Huang C.-S. (2017). Polymorphisms of ESR1, UGT1A1, HCN1, MAP3K1 and CYP2B6 Are Associated with the Prognosis of Hormone Receptor-Positive Early Breast Cancer. Oncotarget.

[75] Nowell S.A., Ahn J., Rae J.M., Scheys J.O., Trovato A., Sweeney C., MacLeod S.L., Kadlubar F.F., Ambrosone C.B. (2005). Association of Genetic Variation in Tamoxifen-Metabolizing Enzymes with Overall Survival and Recurrence of Disease in Breast Cancer Patients. Breast Cancer Res. Treat..

[76] Nowell S., Sweeney C., Winters M., Stone A., Lang N.P., Hutchins L.F., Kadlubar F.F., Ambrosone C.B. (2002). Association between Sulfotransferase 1A1 Genotype and Survival of Breast Cancer Patients Receiving Tamoxifen Therapy. J. Natl. Cancer Inst..

[77] Fernández-Santander A., Gaibar M., Novillo A., Romero-Lorca A., Rubio M., Chicharro L.M., Tejerina A., Bandrés F. (2013). Relationship between Genotypes Sult1a2 and Cyp2d6 and Tamoxifen Metabolism in Breast Cancer Patients. PLoS ONE.

[78] Miranda C., Galleguillos M., Torres R., Tardón K., Cáceres D.D., Lee K., Redal M.A., Varela N.M., Quiñones L.A. (2021). Preliminary Pharmacogenomic-Based Predictive Models of Tamoxifen Response in Hormone-Dependent Chilean Breast Cancer Patients. Front. Pharmacol..

[79] Hoffmeyer S., Burk O., von Richter O., Arnold H.P., Brockmöller J., Johne A., Cascorbi I., Gerloff T., Roots I., Eichelbaum M. (2000). Functional Polymorphisms of the Human Multidrug-Resistance Gene: Multiple Sequence Variations and Correlation of One Allele with P-Glycoprotein Expression and Activity in Vivo. Proc. Natl. Acad. Sci. USA.

[80] Taheri M., Mahjoubi F., Omranipour R. (2010). Effect of MDR1 Polymorphism on Multidrug Resistance Expression in Breast Cancer Patients. Genet. Mol. Res. GMR.

[81] Sensorn I., Sirachainan E., Chamnanphon M., Pasomsub E., Trachu N., Supavilai P., Sukasem C., Pinthong D. (2013). Association of CYP3A4/5, ABCB1 and ABCC2 Polymorphisms and Clinical Outcomes of Thai Breast Cancer Patients Treated with Ta-moxifen. Pharmacogenom. Pers. Med..

[82] Zhang X., Pu Z., Ge J., Shen J., Yuan X., Xie H. (2015). Association of CYP2D6*10, OATP1B1 A388G, and OATP1B1 T521C Pol-ymorphisms and Overall Survival of Breast Cancer Patients after Tamoxifen Therapy. Med. Sci. Monit. Int. Med. J. Exp. Clin. Res..

[83] Gao C.-M., Pu Z., He C., Liang D., Jia Y., Yuan X., Wang G., Xie H. (2017). Effect of OATP1B1 Genetic Polymorphism on the Uptake of Tamoxifen and Its Metabolite, Endoxifen. Oncol. Rep..

[84] Pu Z., Zhang X., Chen Q., Yuan X., Xie H. (2015). Establishment of an Expression Platform of OATP1B1 388GG and 521CC Genetic Polymorphism and the Therapeutic Effect of Tamoxifen in MCF-7 Cells. Oncol. Rep..

[85] Keller D.N., Medwid S.J., Ross C.D., Wigle T.J., Kim R.B. (2023). Impact of Organic Anion Transporting Polypeptide, P-Glycoprotein, and Breast Cancer Resistance Protein Transporters on Observed Tamoxifen and Endoxifen Concentration and Adverse Effects. Pharmacogenet. Genom..

[86] Sanchez-Spitman A.B., Dezentjé V.O., Swen J.J., Moes D.J.A.R., Gelderblom H., Guchelaar H.-J. (2018). Genetic Polymorphisms of 3′-Untranslated Region of SULT1A1 and Their Impact on Tamoxifen Metabolism and Efficacy. Breast Cancer Res. Treat..

[87] Forat-Yazdi M., Jafari M., Kargar S., Abolbaghaei S.M., Nasiri R., Farahnak S., Foroughi E., Neamatzadeh H. (2017). Association between SULT1A1 Arg213His (rs9282861) Polymorphism and Risk of Breast Cancer: A Systematic Review and Meta-Analysis. J. Res. Health Sci..

[88] El Daibani A.A., Alherz F.A., Abunnaja M.S., Bairam A.F., Rasool M.I., Kurogi K., Liu M.-C. (2021). Impact of Human SULT1E1 Polymorphisms on the Sulfation of 17β-Estradiol, 4-Hydroxytamoxifen, and Diethylstilbestrol by SULT1E1 Allozymes. Eur. J. Drug Metab. Pharmacokinet..

[89] Zhou X., Zheng Z., Xu C., Wang J., Min M., Zhao Y., Wang X., Gong Y., Yin J., Guo M. (2017). Disturbance of mammary UDP-glucuronosyltransferase represses estrogen metabolism and exacerbates experimental breast Cancer. J. Pharm. Sci..

[90] Romero-Lorca A., Novillo A., Gaibar M., Bandrés F., Fernández-Santander A. (2015). Impacts of the Glucuronidase Genotypes UGT1A4, UGT2B7, UGT2B15 and UGT2B17 on Tamoxifen Metabolism in Breast Cancer Patients. PLoS ONE.

[91] Ahmed J.H., Makonnen E., Fotoohi A., Aseffa A., Howe R., Aklillu E. (2019). CYP2D6 Genotype Predicts Plasma Concentrations of Tamoxifen Metabolites in Ethiopian Breast Cancer Patients. Cancers.

[92] Lan B., Ma F., Han M., Chen S., Wang W., Li Q., Fan Y., Luo Y., Cai R., Wang J. (2019). The Effect of Polymorphism in UGT1A4 on Clinical Outcomes of Adjuvant Tamoxifen Therapy for Patients With Breast Cancer in China. Clin. Breast Cancer.

[93] Li J., Bluth M.H. (2011). Pharmacogenomics of Drug Metabolizing Enzymes and Transporters: Implications for Cancer Therapy. Pharmacogenom. Pers. Med..

[94] Teft W.A., Mansell S.E., Kim R.B. (2011). Endoxifen, the Active Metabolite of Tamoxifen, Is a Substrate of the Efflux Transporter P-Glycoprotein (Multidrug Resistance 1). Drug Metab. Dispos. Biol. Fate Chem..

[95] Iusuf D., Teunissen S.F., Wagenaar E., Rosing H., Beijnen J.H., Schinkel A.H. (2011). P-Glycoprotein (ABCB1) Transports the Primary Active Tamoxifen Metabolites Endoxifen and 4-Hydroxytamoxifen and Restricts Their Brain Penetration. J. Pharmacol. Exp. Ther..

[96] McLaughlin A.M., Helland T., Klima F., Koolen S.L.W., van Schaik R.H.N., Mathijssen R.H.J., Neven P., Swen J.J., Guchelaar H.-J., Dalenc F. (2024). CYP2D6 Endoxifen Percentage Activity Model (CEPAM) Consortium. Nonlinear Mixed-Effects Model of Z-Endoxifen Concentrations in Tamoxifen-Treated Patients from the CEPAM Cohort. Clin. Pharmacol. Ther..

[97] Sim S., Mueller-Schoell A., Klopp-Schulze L., Schroth W., Mürdter T., Michelet R., Brauch H., Huisinga W., Joerger M., Neven P. (2020). Obesity Alters Endoxifen Plasma Levels in Young Breast Cancer Patients: A Pharmacometric Simulation Approach. Clin. Pharmacol. Ther..

[98] Smith D.A., Sadler M.C., Altman R.B. (2023). Promises and Challenges in Pharmacoepigenetics. Camb. Prism. Precis. Med..

[99] Woolpert K.M., Ahern T.P., Baurley J.W., Maliniak M.L., Damkier P., Kjærsgaard A., Collin L.J., Hamilton-Dutoit S., Tramm T., Ejlertsen B. (2025). Genetic variants in tamoxifen metabolism and early treatment discontinuation among premenopausal breast cancer patients. Breast Cancer Res. Treat..

[100] Antunes M.V., da Fontoura Timm T.A., de Oliveira V., Staudt D.E., Raymundo S., Gössling G., Biazús J.V., Cavalheiro J.A., Rosa D.D., Wallemacq P. (2015). Influence of CYP2D6 and CYP3A4 Phenotypes, Drug Interactions, and Vitamin D Status on Tamoxifen Biotransformation. Ther. Drug Monit..

[101] Wegman P., Vainikka L., Stål O., Nordenskjöld B., Skoog L., Rutqvist L.-E., Wingren S. (2005). Genotype of Metabolic Enzymes and the Benefit of Tamoxifen in Postmenopausal Breast Cancer Patients. Breast Cancer Res..

[102] Sim S., Lövrot J., Lindh J.D., Bergh J., Xie H. (2018). Effect of CYP2C19 and CYP2D6 Genotype on Tamoxifen Treatment Outcome Indicates Endogenous and Exogenous Interplay. Pharmacogenomics.

[103] Teh L.K., Mohamed N.I., Salleh M.Z., Rohaizak M., Shahrun N.S., Saladina J.J., Shia J.K.S., Roslan H., Sood S., Rajoo T.S. (2012). The Risk of Recurrence in Breast Cancer Patients Treated with Tamoxifen: Polymorphisms of CYP2D6 and ABCB1. AAPS J..

[104] Sensorn I., Sukasem C., Sirachainan E., Chamnanphon M., Pasomsub E., Trachu N., Supavilai P., Pinthong D., Wongwaisayawan S. (2016). ABCB1 and ABCC2 and the Risk of Distant Metastasis in Thai Breast Cancer Patients Treated with Tamoxifen. OncoTargets Ther..

[105] Ashcraft K., Grande K., Bristow S.L., Moyer N., Schmidlen T., Moretz C., Wick J.A., Blaxall B.C. (2022). Validation of Pharmacogenomic Interaction Probability (PIP) Scores in Predicting Drug–Gene, Drug–Drug–Gene, and Drug–Gene–Gene Interaction Risks in a Large Patient Population. J. Pers. Med..

[106] Javan Biparva A., Raoofi S., Rafiei S., Masoumi M., Doustmehraban M., Bagheribayati F., Vaziri Shahrebabak E.S., Noorani Mejareh Z., Khani S., Abdollahi B. (2023). Global Depression in Breast Cancer Patients: Systematic Review and Meta-Analysis. PLoS ONE.

[107] Klomp S.D., Manson M.L., Guchelaar H.-J., Swen J.J. (2020). Phenoconversion of Cytochrome P450 Metabolism: A Systematic Review. J. Clin. Med..

[108] Mostafa S., Kirkpatrick C.M.J., Byron K., Sheffield L. (2019). An Analysis of Allele, Genotype and Phenotype Frequencies, Actionable Pharmacogenomic (PGx) Variants and Phenoconversion in 5408 Australian Patients Genotyped for CYP2D6, CYP2C19, CYP2C9 and VKORC1 Genes. J. Neural Transm. Vienna Austria 1996.

[109] Bousman C.A., Stevenson J.M., Ramsey L.B., Sangkuhl K., Hicks J.K., Strawn J.R., Singh A.B., Ruaño G., Mueller D.J., Tsermpini E.E. (2023). Clinical Pharmacogenetics Implementation Consortium (CPIC) Guideline for CYP2D6, CYP2C19, CYP2B6, SLC6A4, and HTR2A Genotypes and Serotonin Reuptake Inhibitor Antidepressants. Clin. Pharmacol. Ther..

[110] Borges S., Desta Z., Li L., Skaar T.C., Ward B.A., Nguyen A., Jin Y., Storniolo A.M., Nikoloff D.M., Wu L. (2006). Quantitative Effect of CYP2D6 Genotype and Inhibitors on Tamoxifen Metabolism: Implication for Optimization of Breast Cancer Treatment. Clin. Pharmacol. Ther..

[111] Klopp-Schulze L., Mueller-Schoell A., Neven P., Koolen S.L.W., Mathijssen R.H.J., Joerger M., Kloft C. (2020). Integrated Data Analysis of Six Clinical Studies Points Toward Model-Informed Precision Dosing of Tamoxifen. Front. Pharmacol..

[112] Binkhorst L., Bannink M., de Bruijn P., Ruit J., Droogendijk H., van Alphen R.J., den Boer T.D., Lam M.H., Jager A., van Gelder T. (2016). Augmentation of Endoxifen Exposure in Tamoxifen-Treated Women Following SSRI Switch. Clin. Pharmacokinet..

[113] Borges S., Desta Z., Jin Y., Faouzi A., Robarge J.D., Philip S., Nguyen A., Stearns V., Hayes D., Rae J.M. (2010). A Composite Functional Genetic and Co-Medication CYP2D6 Activity Score in Predicting Tamoxifen Drug Exposure Among Breast Cancer Patients. J. Clin. Pharmacol..

[114] Buck S.A.J., Braal C.L., Hofman M.M., Oomen-de Hoop E., de Bruijn P., Ghobadi Moghaddam-Helmantel I.M., Hussaarts K.G.A.M., Vastbinder M.B., van Rossum-Schornagel Q.C., van Schaik R.H.N. (2022). Influence of Probenecid on Endoxifen Systemic Exposure in Breast Cancer Patients on Adjuvant Tamoxifen Treatment. Ther. Adv. Med. Oncol..

[115] Henderson S.L., Teft W.A., Kim R.B. (2016). Profound Reduction in Tamoxifen Active Metabolite Endoxifen in a Breast Cancer Patient Treated with Rifampin Prior to Initiation of an Anti-TNFα Biologic for Ulcerative Colitis: A Case Report. BMC Cancer.

[116] Chiwambutsa S.M., Ayeni O., Kapungu N., Kanji C., Thelingwani R., Chen W.C., Mokone D.H., O’Neil D.S., Neugut A.I., Jacobson J.S. (2023). Effects of Genetic Polymorphisms of Drug Metabolizing Enzymes and Co-Medications on Tamoxifen Metabolism in Black South African Women with Breast Cancer. Clin. Pharmacol. Ther..

[117] Puszkiel A., Arellano C., Vachoux C., Evrard A., Le Morvan V., Boyer J.-C., Robert J., Delmas C., Dalenc F., Debled M. (2019). Factors Affecting Tamoxifen Metabolism in Patients with Breast Cancer: Preliminary Results of the French PHACS Study. Clin. Pharmacol. Ther..

[118] Abdullah-Koolmees H., van Keulen A.M., Nijenhuis M., Deneer V.H.M. (2021). Pharmacogenetics Guidelines: Overview and Comparison of the DPWG, CPIC, CPNDS, and RNPGx Guidelines. Front. Pharmacol..

[119] Drug Label Information and Legend. https://www.pharmgkb.org/page/drugLabelLegend.

[120] Koutsilieri S., Tzioufa F., Sismanoglou D.-C., Patrinos G.P. (2020). Unveiling the Guidance Heterogeneity for Genome-Informed Drug Treatment Interventions among Regulatory Bodies and Research Consortia. Pharmacol. Res..

[121] Shekhani R., Steinacher L., Swen J.J., Ingelman-Sundberg M. (2020). Evaluation of Current Regulation and Guidelines of Pharmacogenomic Drug Labels: Opportunities for Improvements. Clin. Pharmacol. Ther..

[122] Ingelman-Sundberg M. (2024). Pharmacogenomic Prescribing Guidelines: Are They Always Useful?. Clin. Pharmacol. Ther..

[123] Kaur G., Nwabufo C.K. (2024). Healthcare Provider and Patient Perspectives on the Implementation of Pharmacogenetic-Guided Treatment in Routine Clinical Practice. Pharmacogenet. Genom..

[124] Klein C.J., Gong L., Caudle K.E., Naik H., Empey P.E., Hoffman J.M., Scherer S., Iwuchukwu O.F., Gregornik D., Monte A.A. (2025). Toward an Integrated Resource for Pharmacogenomics (PGx): Survey Findings from the Genomic Medicine Communities. Genet. Med..

[125] Morris S.A., Alsaidi A.T., Verbyla A., Cruz A., Macfarlane C., Bauer J., Patel J.N. (2022). Cost Effectiveness of Pharmacogenetic Testing for Drugs with Clinical Pharmacogenetics Implementation Consortium (CPIC) Guidelines: A Systematic Review. Clin. Pharmacol. Ther..

[126] Matey E.T., Ragan A.K., Oyen L.J., Vitek C.R., Aoudia S.L., Ragab A.K., Fee-Schroeder K.C., Black J.L., Moyer A.M., Nicholson W.T. (2022). Nine-Gene Pharmacogenomics Profile Service: The Mayo Clinic Experience. Pharmacogenom. J..

[127] Swen J.J., van der Wouden C.H., Manson L.E., Abdullah-Koolmees H., Blagec K., Blagus T., Böhringer S., Cambon-Thomsen A., Cecchin E., Cheung K.-C. (2023). A 12-Gene Pharmacogenetic Panel to Prevent Adverse Drug Reactions: An Open-Label, Multicentre, Controlled, Cluster-Randomised Crossover Implementation Study. Lancet.

[128] Lerena A., Peñas-LLedó E., de Andrés F., Mata-Martín C., Sánchez C.L., Pijierro A., Cobaleda J. (2020). Clinical Implementation of Pharmacogenetics and Personalized Drug Prescription Based on E-Health: The MedeA Initiative. Drug Metab. Pers. Ther..

[129] McDermott J.H., Tsakiroglou M., Newman W.G., Pirmohamed M. (2025). Pharmacogenomics in the UK National Health Service: Progress towards Implementation. Br. J. Clin. Pharmacol..

[130] van der Wouden C.H., van Rhenen M.H., Jama W.O.M., Ingelman-Sundberg M., Lauschke V.M., Konta L., Schwab M., Swen J.J., Guchelaar H. (2019). Development of the PGx-Passport: A Panel of Actionable Germline Genetic Variants for Pre-Emptive Pharmacogenetic Testing. Clin. Pharmacol. Ther..

[131] Storelli F., Matthey A., Lenglet S., Thomas A., Desmeules J., Daali Y. (2018). Impact of CYP2D6 Functional Allelic Variations on Phenoconversion and Drug-Drug Interactions. Clin. Pharmacol. Ther..

[132] Storelli F., Samer C., Reny J.-L., Desmeules J., Daali Y. (2018). Complex Drug-Drug-Gene-Disease Interactions Involving Cytochromes P450: Systematic Review of Published Case Reports and Clinical Perspectives. Clin. Pharmacokinet..

[133] Lenoir C., Rollason V., Desmeules J.A., Samer C.F. (2021). Influence of Inflammation on Cytochromes P450 Activity in Adults: A Systematic Review of the Literature. Front. Pharmacol..

[134] Braal C.L., Jager A., Hoop E.O., Westenberg J.D., Lommen K.M.W.T., de Bruijn P., Vastbinder M.B., van Rossum-Schornagel Q.C., Thijs-Visser M.F., van Alphen R.J. (2022). Therapeutic Drug Monitoring of Endoxifen for Tamoxifen Precision Dosing: Feasible in Patients with Hormone-Sensitive Breast Cancer. Clin. Pharmacokinet..

[135] Sanchez-Spitman A.B., Moes D.-J.A.R., Swen J.J., Dezentjé V.O., Lambrechts D., Neven P., Gelderblom H., Guchelaar H.-J. (2020). Exposure-Response Analysis of Endoxifen Serum Concentrations in Early-Breast Cancer. Cancer Chemother. Pharmacol..

[136] Braal C.L., Kleijburg A., Jager A., Koolen S.L.W., Mathijssen R.H.J., Corro Ramos I., Wetzelaer P., Uyl-de Groot C.A. (2022). Therapeutic Drug Monitoring-Guided Adjuvant Tamoxifen Dosing in Patients with Early Breast Cancer: A Cost-Effectiveness Analysis from the Prospective TOTAM Trial. Clin. Drug Investig..

[137] Lee C.I., Low S.K., Maldonado R., Fox P., Balakrishnar B., Coulter S., de Bruijn P., Koolen S.L.W., Gao B., Lynch J. (2020). Simplified Phenotyping of CYP2D6 for Tamoxifen Treatment Using the N-Desmethyl-Tamoxifen/Endoxifen Ratio. Breast.

[138] Gusella M., Pasini F., Corso B., Bertolaso L., De Rosa G., Falci C., Modena Y., Barile C., Da Corte Z.D., Fraccon A. (2020). Predicting steady-state endoxifen plasma concentrations in breast cancer patients by CYP2D6 genotyping or phenotyping. Which approach is more reliable?. Pharmacol. Res. Perspect..

[139] Opdam F.L., Dezentje V.O., den Hartigh J., Modak A.S., Vree R., Batman E., Smorenburg C.H., Nortier J.W.R., Gelderblom H., Guchelaar H.-J. (2013). The Use of the 13C-Dextromethorphan Breath Test for Phenotyping CYP2D6 in Breast Cancer Patients Using Tamoxifen: Association with CYP2D6 Genotype and Serum Endoxifen Levels. Cancer Chemother. Pharmacol..

[140] Frye R.F., Matzke G.R., Adedoyin A., Porter J.A., Branch R.A. (1997). Validation of the Five-Drug “Pittsburgh Cocktail” Approach for Assessment of Selective Regulation of Drug-Metabolizing Enzymes. Clin. Pharmacol. Ther..

[141] Ing Lorenzini K., Desmeules J., Rollason V., Bertin S., Besson M., Daali Y., Samer C.F. (2021). CYP450 Genotype—Phenotype Concordance Using the Geneva Micrococktail in a Clinical Setting. Front. Pharmacol..

[142] Suenderhauf C., Berger B., Puchkov M., Schmid Y., Müller S., Huwyler J., Haschke M., Krähenbühl S., Duthaler U. (2020). Pharmacokinetics and phenotyping properties of the Basel phenotyping cocktail combination capsule in healthy male adults. Br. J. Clin. Pharmacol..

[143] Magliocco G., Desmeules J., Matthey A., Quirós-Guerrero L.M., Bararpour N., Joye T., Marcourt L., Queiroz E.F., Wolfender J.-L., Gloor Y. (2021). Metabolomics Reveals Biomarkers in Human Urine and Plasma to Predict Cytochrome P450 2D6 (CYP2D6) Activity. Br. J. Pharmacol..

[144] Wollmann B.M., Størset E., Kringen M.K., Molden E., Smith R.L. (2023). Prediction of CYP2D6 Poor Metabolizers by Measurements of Solanidine and Metabolites—A Study in 839 Patients with Known CYP2D6 Genotype. Eur. J. Clin. Pharmacol..

[145] Oda A., Suzuki Y., Sato H., Koyama T., Nakatochi M., Momozawa Y., Tanaka R., Ono H., Tatsuta R., Ando T. (2024). Evaluation of the Usefulness of Plasma 4β-Hydroxycholesterol Concentration Normalized by 4α-Hydroxycholesterol for Accurate CYP3A Phenotyping. Clin. Transl. Sci..

[146] Taya Y., Mizunaga M., Nakao S., Jutanom M., Shimizu N., Nomura Y., Nakagawa K. (2023). Clinical Evaluation Based on a New Approach to Improve the Accuracy of 4β-Hydroxycholesterol Measurement as a Biomarker of CYP3A4 Activity. Molecules.

